# Bilateral Metastatic Axillary Adenopathy Revealing Serous Carcinoma of the Ovary: A Case Report and Review of the Literature

**DOI:** 10.7759/cureus.89899

**Published:** 2025-08-12

**Authors:** Moctar Noufou Fodiya, Sara Chater, Kenza Bentalha, Bouchra Kouhkouh, Safia Gmih, Habib Dato Outban, Fatima Zahra Belkouchi, Sanae Amalik, Fouad Tijami, Hafid Hachi, Ismail Boujida, Hanane Inrhaoun, Nezha Elbahaoui

**Affiliations:** 1 Gyneco-Breast Surgery Unit, Sidi Mohamed Ben Abdellah National Institute of Oncology, Ibn Sina University Hospital, Mohammed V University, Rabat, MAR; 2 Obstetrics and Gynecology, Souissi Maternity Hospital, Ibn Sina University Hospital, Mohammed V University, Rabat, MAR; 3 Imaging Department, Sidi Mohamed Ben Abdellah National Institute of Oncology, Ibn Sina University Hospital, Mohammed V University, Rabat, MAR; 4 Anatomical Pathology Laboratory, Sidi Mohamed Ben Abdellah National Institute of Oncology, Ibn Sina University Hospital, Mohammed V University, Rabat, MAR; 5 Medical Oncology Department, Sidi Mohamed Ben Abdellah National Institute of Oncology, Ibn Sina University Hospital, Mohammed V University, Rabat, MAR

**Keywords:** axillary lymph node metastasis, case report, cytoreductive surgery (crs), distant metastasis, ovarian serous carcinoma

## Abstract

Ovarian cancer represents a major public health problem. It is the deadliest gynecological cancer with a high metastatic potential. Unilateral axillary lymph node metastases are rarely seen in ovarian cancer, and bilateral lymph node metastases are exceptional.

We report a case of a 61-year-old female patient presenting with bilateral axillary lymphadenopathy secondary to high-grade serous carcinoma of the right ovary. Treatment consisted of lymph node excision, adnexectomy, cytoreduction, and chemotherapy. Diagnostic, therapeutic, and prognostic aspects are discussed through a literature review.

The axillary localization of ovarian cancer, although rare, can be observed, especially in the postmenopausal period. Treatment is based on cytoreductive surgery but can be supplemented by neoadjuvant chemotherapy.

## Introduction

Ovarian cancer is a public health problem and is the deadliest cancer of all gynecological cancers [1]. It has an average five-year survival rate of 31%, the lowest of all gynecological cancers, followed by vaginal cancer at 44.8% [2]. They are dominated by epithelial tumors, the most common of which is high-grade serous carcinoma. The latter is most often diagnosed in an advanced stage with a high metastatic potential [3]. Ovarian cancer metastasizes primarily intraperitoneally (Douglas pouch, extracurricular aligners, liver, splenic capsules, small intestine omentum, or mesentery) or distant (liver, lung, or pleura) [4].

Isolated axillary lymph node metastases are rare, with 33 cases reported in the literature, and bilateral forms are even rarer, with only two cases reported in the literature [5,6].

We report a case of serous carcinoma of the right ovary in a 61-year-old woman discovered by bilateral axillary lymphadenopathy.

## Case presentation

Patient history

The patient was a 61-year-old woman with no significant pathological history, such as cancer or other chronic diseases, who was referred to our structure (the Gyneco-Breast Surgery Unit of the Rabat National Institute of Oncology) for a fortuitous discovery following a self-examination of two bilateral axillary masses, one on the right and one on the left.

Clinical findings

**Figure 1:**
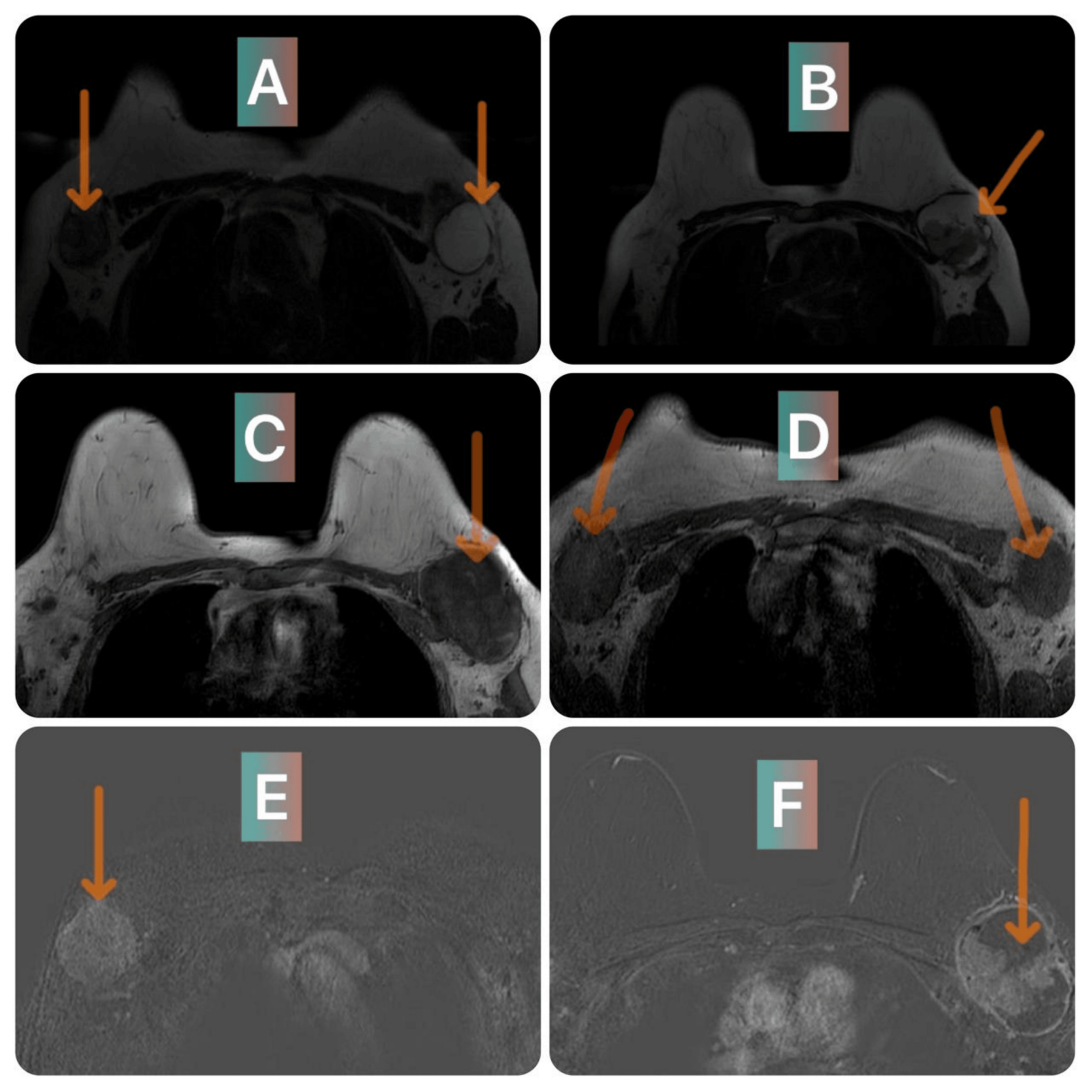
Breast MRI. T2-weighted (A and B) and T1-weighted (C and D) images with subtraction sequence (E) showing two masses in the axillary extensions, with irregular contours, containing a few liquid areas of necrosis, intermediate T2 heterogeneous signal, T1 hyposignal, restrictive to diffusion, and enhanced after injection of gadolinium (F).

The patient was received with axillary and breast ultrasound, which revealed a hypoechoic mass of the left axillary extension with foci of necrosis and the presence above of small homogeneous lymph nodes measuring 52 x 41 x 57 mm, with a strong suspicion of malignancy; on the right there was no suspicion of signs of malignancy, so there was no suspicious mass in either breast. On clinical examination, we found two axillary masses that were hard, fixed, and had irregular contours, measuring 5 cm and 8 cm, respectively, on the right and left sides. In front of the axillary masses and the absence of a breast mass, we performed a breast MRI, which objectified two masses at the level of the right and left axillary extensions with a strong suspicion of malignancy (Figure 1).

**Figure 2:**
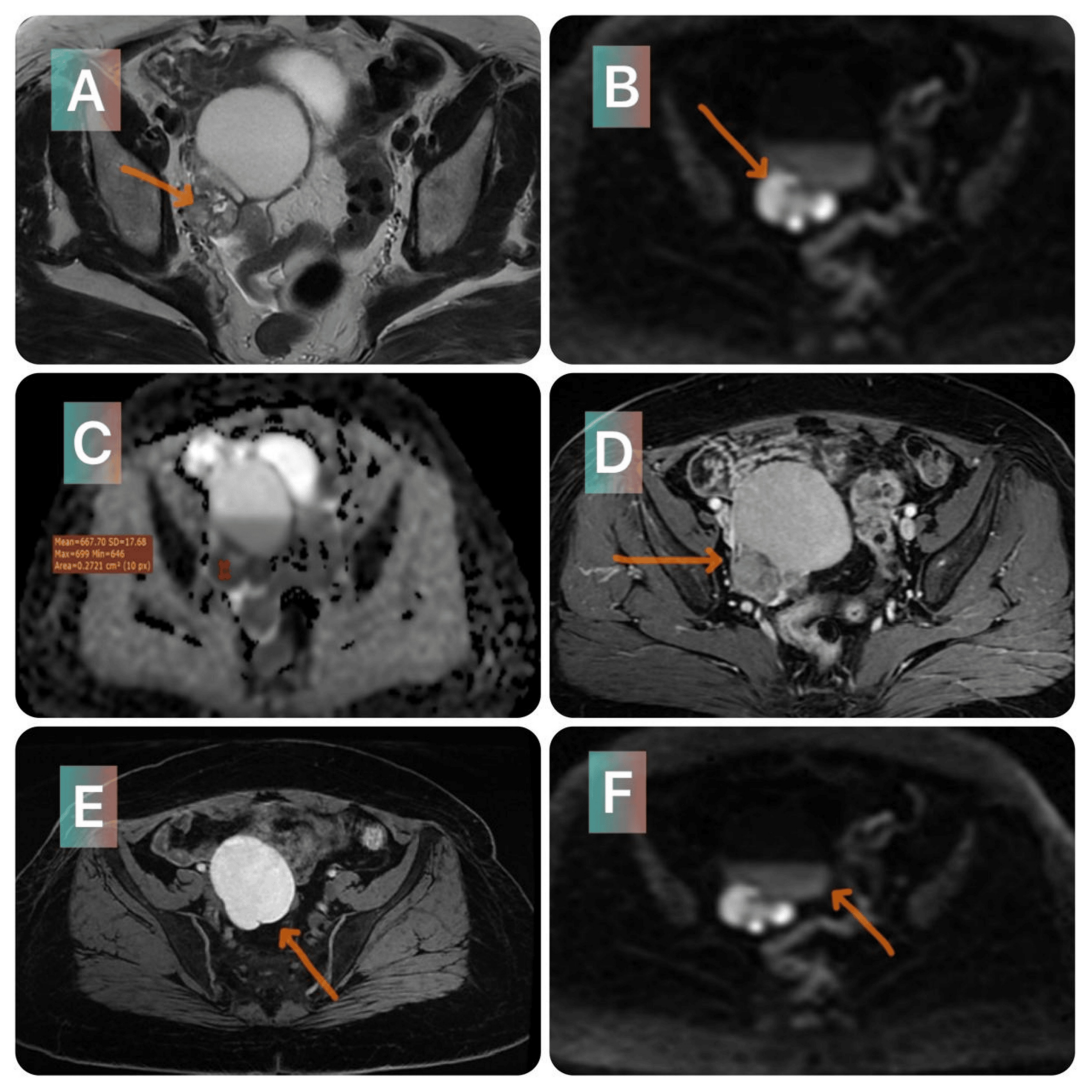
Pelvic MRI. MRI in axial T2 sequence, without fat saturation before and after gadolinium injection + diffusion sequences with apparent diffusion coefficient (ADC) mapping: Solidocystic mass, with tissue portion in intermediate T2 signal (A), diffusion hyper signal with drop in ADC (B and C), enhanced after gadolinium injection (D). T1 fat-saturated and diffusion sequence: Multiloculated fluid portion, with two thin-walled cubicles, with hemorrhagic content in hypersignal T1 (E), containing a liquid-liquid level visible at the sequence diffusion (F).

Microbiopsies were performed, and the histopathological results were inconclusive. According to the microbiopsies, the one on the right was of neoplastic origin, while the one on the left was fibrosis tissue free of tumor lesions. A second biopsy of the left axillary mass was performed, which confirmed the diagnosis of solid papillary carcinoma. A thoraco-abdomino-pelvic CT scan was ordered as part of the extension work-up. It revealed bilateral heterogeneous axillary lymphadenopathy associated with a suspicious right adnexal mass, with no other secondary lesions. We completed the assessment with a pelvic MRI, which was in favor of a right ovarian mass classified O-RADS IV (Ovarian-Adnexal Reporting Data System), suggestive of an epithelial tumor without excluding an endometrial tumor or a clear cell tumor (Figure 2).

Tumor markers were performed, the results of which can be found in Table 1.

**Table 1:** Tumor marker values. CA 125: cancer antigen 125; CA 15-3: cancer antigen 15-3; U/mL: units per milliliter.

Tumor markers	Result	Reference values
CA 125	1000 U/mL	<35 U/mL
CA 15-3	44.31 U/mL	<30 U/mL

Given these results, and after consultation, we decided to perform a diagnostic right adnexectomy associated with a bilateral axillary adenectomy. The patient underwent a diagnostic laparotomy via a median incision under the umbilicus, revealing a pseudocystic right ovarian mass in close contact with the rectosigmoidal hinge, with no peritoneal carcinosis. A right adnexectomy was performed with peritoneal staging. Second stage of surgical exploration was followed by a bilateral axillary incision, resulting in a diagnostic bilateral adenectomy, with no residue left.

**Figure 3:**
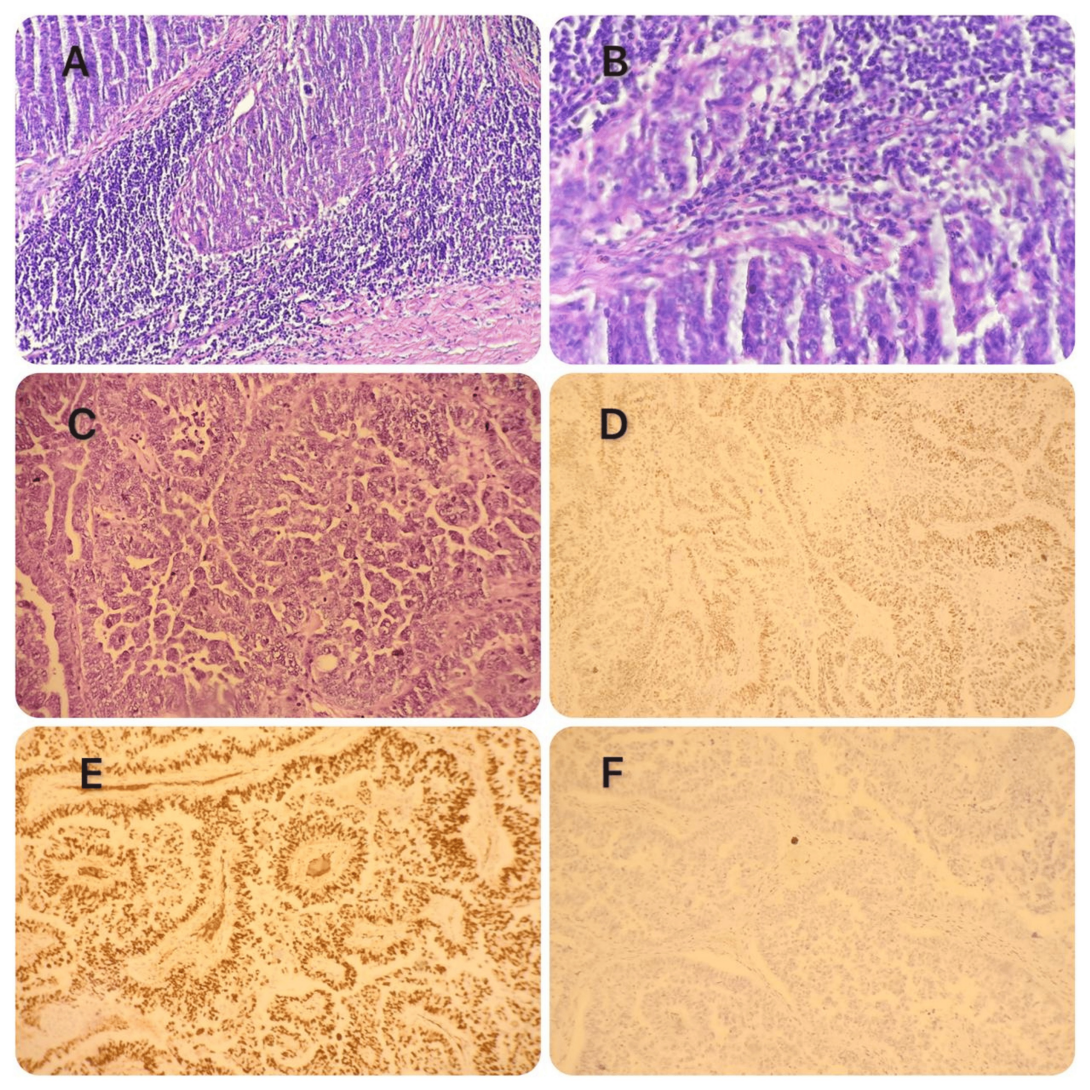
Histological images. A: Metastatic involvement of the right axillary lymph node by high-grade serous carcinoma of the ovary (HE, x200). B: Metastatic involvement of the left axillary lymph node by high-grade serous carcinoma of the ovary (HE, x200). C: Papillary architecture formed by atypical cells with large pleomorphic nuclei and prominent nucleoli (high-grade serous carcinoma of the ovary) (HE, x400). D: Positive immunohistochemical staining of tumor cells for estrogen receptor (ER) (IHC, x200). E: Positive immunohistochemical staining of tumor cells for anti-WT1 protein (IHC, x200). F: Tumor cells showing absence of p53 expression by immunohistochemistry (IHC, x200). HE: hematoxylin and eosin; IHC: immunohistochemistry.

The histopathological examination, followed by an immunohistochemical complement of the right appendectomy operative specimen, was in favor of high-grade serous carcinoma of the ovary. Those of the left and right lymphadenopathy were respectively in favor of the left and right lymph node locations of a high-grade serous carcinoma of known ovarian origin (Figure 3).

A PET scan was requested postoperatively, revealing no fixation anomalies in the entire volume explored.

In light of all these elements, the case was discussed in a specialized institutional ovarian tumor multidisciplinary consultation meeting (MCM), and the decision was to proceed with complete cytoreduction surgery, followed by adjuvant chemotherapy.

Therapeutic intervention

The patient underwent a new operation with a midline xipho-pubic incision. Intraoperative exploration found peritoneal carcinomatosis with centimetric nodules on the right diaphragmatic dome peritoneum, multiple millimetric nodules on the greater omentum, and similar lesions on the anterior surface of the sigmoid colon. The Peritoneal Carcinomatosis Index (PCI) was estimated at 5 out of 39. She underwent complete cytoreduction with total hysterectomy, appendectomy, infragastric omentectomy, resection of a patch of the right diaphragmatic cupola, bilateral pelvic and lumbar-aortic dissection, and resection of nodules suspected of carcinosis. Pathological examination of the various surgical specimens revealed no metastases, except for a poorly differentiated, high-grade carcinomatous process located in the diaphragm. The case was discussed again at the MCM, where the decision was taken to perform neoadjuvant chemotherapy.

Follow-up

The patient developed a Clavien-Dindo grade I postoperative complication consisting of a superficial wound infection, which was managed with twice-daily local wound care, resulting in a favorable clinical outcome [7]. Oral intake was authorized on the first postoperative day (POD), and the patient was discharged on POD 10. The patient was referred to a medical oncology department, where she is currently undergoing a chemotherapy protocol based on paclitaxel and carboplatin.

## Discussion

Ovarian cancer is a major public health problem. It is the deadliest form of gynecologic cancer, the seventh most common form of cancer, and the eighth leading cause of cancer death in women worldwide [1]. The epithelium is the outer layer that lines the ovaries; it is the cause of 90% of ovarian tumors, whether benign or malignant. There are seven histological subtypes: serous, endometrioid, mucinous, clear cell, transitional cell, undifferentiated, and mixed. Serous subtypes are the most common, accounting for 50% to 70%, followed by the endometrioid and mucinous subtypes, each at 10% to 25% and 5% to 10%, respectively [8]. Axillary lymph node metastases of ovarian cancer are rare entities, so after conducting an internet search, we identified 33 cases, series, and case reports combined published in 20 articles [4,6]. The bilateral axillary lymph node metastasis depicted in our case is quite rare, with only two cases reported out of the 33 reported in the literature [5,6].

Serous carcinoma of the ovary metastasizes by three routes: either through the transcoelomic route, the most common, which leads to rapid and early dissemination, facilitated by the ascites fluid that surrounds the primary tumor, causing its spread to the peritoneum, omentum, and organs of the peritoneal cavity. This explains its aggressiveness and high metastatic potential [9], or even more, the hematogenous route to the lungs, liver, pleura, or brain. And finally, either by lymphatic track, a rare mode of dissemination of serous carcinoma of the ovary. The mechanism of lymphatic dissemination at a distance, such as in axillary lymph node metastases of ovarian cancer, to which our case is subject, has been described.

Ovarian cancer most frequently manifests as ascites associated with peritoneal carcinomatosis. In this context, tumor cells can spread to the supradiaphragmatic compartment via transdiaphragmatic infiltration. Dissemination from this site can take place via two main lymphatic routes. The first, anterior, is via the prepericardial lymph nodes, which drain either into the internal jugular and subclavian veins or into the subclavian lymph trunk, extending to the axillary lymph nodes. The second, posterior pathway, results from the confluence of infra-diaphragmatic lymphatic vessels and superficial lymphatics below the level of the umbilicus, forming the chyle cistern, from which emerges the thoracic duct, which drains at the junction of the left subclavian vein and the internal jugular vein [10]. Although our patient did not reveal a family history of ovarian cancer, there is a component related to genetic factors. Thus, the risk of developing ovarian cancer in a woman with a first-degree family history is three times higher than in a woman without a family history [11]. As was the case with our 61-year-old patient, ovarian cancer occurs after the age of 40, with more than 90% of cases occurring in epithelial tumors, and the risk increases with age, with a peak in the late 60s [12]. The mode of presentation can be synchronous (simultaneous discovery of ovarian cancer and axillary metastases), as in our case, or the case of recurrence (treated ovarian cancer that recurs by presenting axillary metastases). Of the two cases of bilateral axillary metastases, we have one case of synchronous presentation and one case of recurrence.

Ovarian cancer presents a major diagnostic challenge. This is due not only to the fact that its symptoms are non-specific and often appear at an advanced stage of the disease, but also to the lack of specific early detection, as with breast and cervical cancer.

The treatment is, above all, surgical; it is not limited to lymph node excision, and it must be carcinological. In advanced forms, as in most cases, ovarian carcinoma is diagnosed at an advanced stage, and carcinologic excision, consisting of complete cytoreduction, must be followed by adjuvant chemotherapy. This is the recommended protocol, which improves overall patient follow-up [13]. Often, the therapeutic attitude also depends on the mode of presentation. If it is a synchronous presentation, as in the present case, cytoreduction must be supplemented by adjuvant chemotherapy. This is why in our patient, the first surgery (adnexectomy and excision of lymphadenopathy) was completed by cytoreductive surgery, followed by adjuvant chemotherapy. All decisions were taken at an MCM.

The disease has a poor prognosis, as early diagnosis is rare, and the vast majority of cases are diagnosed at the metastatic stage, as was the case with our patient. This results in a five-year survival rate of around 29.2%, with a recurrence rate of 70-90% within 18 months for advanced forms [14]. According to the recommendations of the Society of Gynecologist Oncologists (SGO) for patients with a history of epithelial ovarian cancer, follow-up consists of a systems examination and a physical examination every three months for the first two years, every four to six months for the third year, every six months for the fourth and fifth years, and every year after five years. The SGO does not recommend vaginal cytology in the case of hysterectomy, nor imaging in the absence of signs, and the dosage of CA-125 is optional. CT and/or PET scan and CA-125 are recommended in the event of recurrence [15].

## Conclusions

Although rare, axillary metastases of ovarian cancer occur mainly in patients aged 60 years and over and are mostly associated with serous carcinoma. They may occur synchronously or in the context of recurrence. Management is based on complete cytoreduction, often supplemented by other therapeutic modalities depending on tumor stage and clinical context. Clinicians should be particularly vigilant, systematically looking for axillary adenopathy in elderly patients with ovarian cancer.
